# Procedural and outcome impact of obesity in cryoballoon versus radiofrequency pulmonary vein isolation in atrial fibrillation patients

**DOI:** 10.1007/s10840-022-01210-3

**Published:** 2022-04-12

**Authors:** Cornelia Scheurlen, Jan-Hendrik van den Bruck, Karlo Filipovic, Jonas Wörmann, Zeynep Arica, Susanne Erlhöfer, Sebastian Dittrich, Jordi Heijman, Jakob Lüker, Daniel Steven, Arian Sultan

**Affiliations:** 1 Department of Electrophysiology, Heart Center, University of Cologne, Kerpener Str. 62, 50937 Köln, Germany; 2 Department of Cardiology, Maastricht University, Maastricht, Netherlands

**Keywords:** Atrial fibrillation, Pulmonary vein isolation, Cryoballoon PVI, Radiofrequency PVI, Obesity, BMI

## Abstract

**Purpose:**

Cryoballoon (CB) ablation and radiofrequency (RF) ablation are the most common techniques for pulmonary vein isolation (PVI) in patients with symptomatic atrial fibrillation (AF). An increasing number of patients undergoing PVI are obese.

To address the paucity of data on outcomes of CB- vs. RF-based PVI in relation to body mass index (BMI) of AF patients.

**Methods:**

All patients undergoing de novo PVI between 01/2018 and 08/2019 at University Hospital Cologne were included in this retrospective analysis. Patients of each group (CB-PVI vs. RF-PVI) were analyzed based on their BMI. Hereafter, procedural characteristics and AF recurrence rate were compared regarding different BMI groups.

**Results:**

A total of 526 patients (62% male, 65±11 years) underwent successful de novo PVI (320 CB and 206 RF). In obese patients, two differences in procedural characteristics were noted: A significantly increased contrast medium volume in CB group and a lower fluoroscopy dose in RF group: contrast medium: CB 50 [40-80] vs. RF 20 [20-30], *p*<0.001; fluoroscopy dose: CB 392.4 [197.9-995.9] vs. RF 282.5 [139.8-507.2], *p*<0.001. The complication rate was equal throughout all BMI groups, regardless of CB or RF usage. For obese patients, a trend toward a higher AF recurrence rate was revealed after RF-PVI as compared to CB-PVI. In line with previous studies, the overall procedure time was significantly shorter with CB-PVI regardless of BMI.

**Conclusion:**

For obese patients, CB-PVI is similarly safe and effective as RF-PVI. The significantly shorter procedure time for CB-PVI may minimize potential obesity-related complications. However, the lower contrast medium quantity and fluoroscopy dose in RF-PVI must be considered. AF recurrence rates were comparable between CB-PVI and RF-PVI.

## Introduction

Atrial fibrillation (AF) is the most common cardiac arrhythmia in adults and is associated with an increased rate of mortality, congestive heart failure and stroke [1, 2].

Pulmonary vein isolation (PVI) is an established treatment option in symptomatic AF patients. The current guidelines favor catheter ablation as a Class I indication in symptomatic patients with drug-refractory AF [1], independent of a paroxysmal (PAF) or persistent (pers AF) character. As a first-line therapy in symptomatic patients, PVI significantly reduces symptoms and AF burden and increases the quality of life [1, 3].

Cryoballoon (CB) ablation and radiofrequency (RF) ablation are currently the most commonly used techniques for PVI [4], and both methods are considered equivalent in current guidelines [1]. Both approaches also showed similar acute success and AF recurrence rates [5].

Although management of AF-promoting comorbidities such as obesity is strongly recommended by the current AF guidelines [1, 6], there are no restrictions for catheter ablation of AF with respect to the body mass index (BMI, defined as a person’s weight in kilograms divided by the square of their height in meters [kg/m^2^]).

Obesity (defined as BMI≥30 kg/m^2^ [7]) is a widespread and rapidly growing healthcare burden and is associated with a higher risk of cardiovascular events as well as complications during interventions such as catheter ablation [8–11]. In particular, deep analgo-sedation in obese patients is associated with a higher risk of sedation-related complications such as hypoxemia, hypotension and aspiration [12, 13]. Indeed, even moderate obesity is a risk factor for aspiration [12]. With increasing BMI, the risk for aspiration is significantly increased and advanced airway management should be considered [12]. Furthermore, obesity is an independent risk factor for AF [1, 4]. Therefore, many patients with an indication for PVI are overweight or obese. Additionally, obesity negatively affects the outcome after an initially successful PVI [14]. The benefits of weight control to reduce post-ablation AF recurrence have previously been demonstrated [6]. However, effective and persistent weight management is challenging and in daily practice a notable proportion of obese patients still undergo PVI. In most clinical trials, BMI was not a selection criterion and obese patients were not well represented. For example, in the FIRE and ICE trial mean BMI in the CB group was 28±4.7 kg/m^2^, and no sub-analysis for different BMI classes was conducted [5]. Thus, despite a rapid increase in patients with AF [1, 2, 15] and concomitant obesity [7], data on the optimal technology for ablation for these patients is still scarce. Therefore, the present study assessed the impact of BMI on procedural characteristics, efficacy and safety, as well as AF recurrence rate during follow-up in CB-PVI vs. RF-PVI.

## Methods

### Study population

All patients undergoing de novo PVI between January 2018 and August 2019 at the University Hospital of Cologne were included in this retrospective analysis. Inclusion criteria were symptomatic AF episodes, age>18 years and having given written informed consent. Patients with prior left atrial ablation were excluded from this study. The data acquisition was performed using *RedCap Database, Nashville, Tennessee, USA*.

Pre-definition of subgroups was based on BMI according to the WHO classification: normal weight BMI<25 kg/m^2^, pre-obesity (preOb) 25-30 kg/m^2^, obesity (Ob) 30-35 kg/m^2^ and severe obesity (sOb) ≥35 kg/m^2^ (combining WHO obesity groups II and III) [7]. We compared CB vs. RF-PVI for efficacy, safety and procedural characteristics (e.g., procedure time, contrast medium volume and fluoroscopy dose) according to BMI subgroups.

The study complied with the Declaration of Helsinki. The local Ethics Committee approved the study protocol, and all included participants signed informed consent to the procedure and the general data processing and analysis.

### Ablation procedure

A preprocedural transesophageal echocardiography was performed to rule out any left atrial appendage or left atrial thrombi irrespective of previous oral anticoagulation for both CB-PVI and RF-PVI groups. Oral anticoagulation was discontinued one day prior to the procedure and continued immediately after the procedure. In patients on vitamin K antagonists, the procedure was performed within a therapeutic international normalized ratio (INR) range of 2.0-3.5. If INR was <2.0, patients received weight adjusted heparin until the day of the procedure. In case INR was >3.5, patients received an adjusted dosage of Konakion (Vitamin K 1) to lower the INR or the procedure was postponed at operators’ discretion. All ablation procedures were performed under deep sedation using propofol, midazolam and fentanyl. A continuous oxygen insufflation and airway protection using oropharyngeal tubes were established.

After obtaining triple, or in case of CB-PVI double, femoral venous access through the right femoral vein and positioning of a decapolar catheter (*Dynamic XT™, large curve 4.0/Decapolar, Boston Scientific, Marlborough, Massachusetts, USA*) in the coronary sinus, a fluoroscopy-guided single transseptal puncture using *TSX™ Fixed Curve Transseptal Sheath and TSX™ Transseptal Needle* (*Boston Scientific, Marlborough, Massachusetts, USA)* was performed. Immediately after successful transseptal puncture, a weight-adjusted heparin bolus was administered, with repeat boluses every 30 minutes targeting an activated clotting time of >300s. For esophageal temperature monitoring, a temperature probe (*S-Cath, Esophageal Temperature Probe, Circa Scientific Inc., Englewood, Colorado, USA*) was orally placed in the esophagus in both groups. Procedure time was defined as skin-to-skin time (groin puncture to groin suture after sheath removal). At the end of the procedure and 2 hours after pericardial effusion was excluded using an echocardiogram. Oral anticoagulation was continued the same day.

#### Cryoballoon pulmonary vein isolation

All CB procedures in this study were performed using the *FlexCath Advance™* and the *Arctic Front Advance Pro™, Medtronic, Dublin, Ireland*. The CB was placed in each PV antrum under fluoroscopy guidance. Optimal PV occlusion by CB was evaluated via contrast medium application. To obtain PV signals and to assess electrical isolation, a decapolar circumferential mapping catheter (*Achieve™ and Achieve Advance™ Mapping Catheters, Medtronic*) was used. The freeze duration was determined by either the observed time to isolation (TTI; in case of visible PV signals) or achieved nadir temperature during the applied freeze [16, 17]. During CB-PVI of the right PVs, continuous phrenic nerve pacing was performed to avoid phrenic nerve palsy. The endpoint for CB procedures was proof of entrance block using the Achieve mapping catheter.

#### Radiofrequency pulmonary vein isolation

All RF-PVI procedures were performed using a three-dimensional mapping system such as *CARTO®3 System by Biosense Webster, Irvine, California, USA* or *EnSite Precision™ Cardiac Mapping System by Abbott, Chicago, Illinois, USA*. When using the *CARTO®3 System,* one of the following mapping and ablation catheters was used at the operator’s discretion: *LASSO® NAV eco catheter D-Type/LASSO 15mm or 20mm, PentaRay® NAV eco high-density mapping catheter D- or F-curve; ThermoCool® SmartTouch SF uni-directional navigation catheter D- or F-type (all Biosense Webster, Irvine, California, USA).* When using the *EnSite Precision™,* one of the following mapping and ablation catheters was used: *Advisor™ FL Sensor Enabled™ D- or F-curve, Advisor™ HD-Grid Sensor Enabled™ D- or F-curve; TactiCath™ Sensor Enabled™ D- or F-curve, FlexAbility™ Sensor Enabled™ D- or F-curve (all Abbott,* Chicago, Illinois, USA).

In all RF-PVI, a contact force catheter was used aiming for a contact force of 5-20g. Energy titration was guided by lesion size index (LSI) or ablation index (AI) depending on the used mapping system, aiming for a target of 5.5 at the anterior and 4.5 at the posterior wall in case of LSI and 550 at the anterior and 400 at the posterior wall for AI. The maximum power was 30-40 watt at operator´s discretion. A point-by-point ablation approach with the superior endpoint of non-excitability along the circumferential ablation line was performed. Non-excitability was evaluated by loss of pace capture along the ablation line [18]. Pulmonary veins were checked for entrance-, if feasible for exit block.

### Clinical follow-up

For all patients, a three- and 12-month post-ablation follow-up visit was scheduled in our out-patient clinic. For detection of AF recurrence, a 24-hour-Holter electrocardiogram (ECG) was performed before each visit. If patients reported symptoms, additional Holter-ECG was obtained. During the follow-up visit, a 12-lead-ECG was recorded and the patient’s history was taken. Arrhythmia recurrence was defined as recurrence of any arrhythmia longer than 30 seconds (AF, AT, atrial flutter) after a 90-day blanking period [19].

### Study endpoints

Study endpoints were differences in procedural characteristics (procedure time, contrast medium and fluoroscopy dose, additional in CB procedures number of freezes and freeze duration) and recurrence rates of any atrial arrhythmia >30 seconds for each ablation technique in the different obesity groups.

### Statistical analysis

Continuous data were shown as mean and standard deviation (SD) and categorial variables as counts and percentages. For skewed data median and IQR were used. Statistical significance was evaluated by students´ t-test, Pearson´s Chi-squared test and Kruskal–Wallis test. A *p*-value <0.05 was considered statistically significant. *P*-values are Bonferroni-corrected. Cox regression was used to evaluate the correlation between different parameters.

## Results

### Study population

**Fig. 1.**
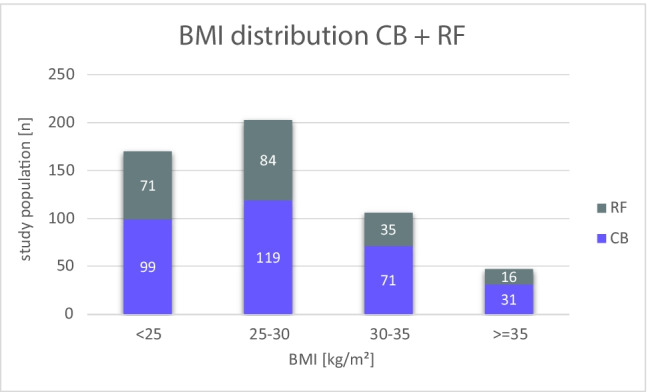
BMI distribution. CB: Cryoballoon-, RF: radiofrequency-pulmonary vein isolation, BMI: body mass index, n: number

In total, 526 de novo PVI were performed successfully: 320 CB-PVI (61%) and 206 RF-PVI (39%). The mean age was 65.3 ± 11.2 years (CB 64.9 ± 11.4, RF 66.0 ± 10.8) and 325 (62%) were male (CB 63%, RF 61%). A total of 312 patients (59%) suffered from PAF and 214 patients (41%) from pers AF. The mean BMI was 27.7 ± 4.8 kg/m^2^ (CB 28.0 ± 4.8, RF 27.3 ± 4.7, *p*=0.10). The BMI distribution for CB group was: 99 (31%), 119 (37%), 71 (22%) and 31 (10%) patients in the normal, preOb, Ob and sOb groups, respectively. For the RF group, this distribution was: 71 (34%) patients NW, 84 (41%) preOb, 35 (17%) Ob and 16 (8%) sOb (Fig. 1). The baseline characteristics are shown in Tables 1 and 2. There were no significant differences in baseline characteristics between patients undergoing CB-PVI vs. RF-PVI (Table 1). However, as expected, several clinical characteristics differ between the different BMI groups. With increasing BMI, the age of patients with AF decreases, but the number of comorbidities, like hypertension, diabetes mellitus and obstructive sleep apnea, increases (Table 2).

**Table 1:** Baseline characteristics in CB and RF group, continuous data are summarized as means ± standard deviation, categorial data are presented as number [percent]

Baseline characteristics	All	CB	RF	*p value*
Patients, n (%)	526	320 (61%)	206 (39%)	
Age [years]	65.3 ± 11.2	64.9 ± 11.4	66.0 ± 10.8	0.92
Male gender, n (%)	325 (62%)	200 (63%)	125 (61%)	0.68
Paroxysmal AF, n (%)	312 (59%)	192 (60%)	120 (58%)	0.69
Persistent AF, n (%)	214 (41%)	128 (40%)	86 (42%)	
BMI [kg/m^2^]	27.7 ± 4.8	28.0 ± 4.8	27.3 ± 4.7	0.10
Comorbidities				
Arterial hypertension, n (%)	368 (70%)	218 (68%)	150 (73%)	0.25
Diabetes mellitus type II, n (%)	50 (10%)	32 (10%)	18 (9%)	0.63
Prior stroke, n (%)	42 (8%)	25 (8%)	17 (8%)	0.86
CAD, n (%)	100 (19%)	63 (20%)	37 (18%)	0.62
OSA, n (%)	33 (6%)	20 (6%)	13 (6%)	0.98
CHA_2_DS_2_-VASc-score	2.5 ± 1.5	2.5 ± 1.6	2.5 ± 1.5	1.00

*CB: Cryoballoon-, RF: radiofrequency-pulmonary vein isolation, n: number, AF: atrial fibrillation, BMI: body mass index,* CAD: coronary artery disease, OSA: obstructive sleep apnea.

**Table 2:** Baseline characteristics in BMI-groups, continuous data are summarized as means ± standard deviation, categorial data are presented as number [percent]

Baseline characteristics	All	BMI<25	BMI 25-30	BMI 25-30	BMI≥35	*p value*
Patients, n (%)	526	170	203	106	47	
Age [years]	65.3 ± 11.2	66.7 ± 11.7	65.7 ± 11.2	64.4 ± 10.2	60.5 ± 9.6	0.006*
Male gender, n (%)	325 (62%)	90 (53%)	146 (72%)	65 (61%)	24 (51%)	0.001*
Paroxysmal AF, n (%)	312 (59%)	117 (69%)	120 (59%)	52 (49%)	23 (49%)	0.004*
Persistent AF, n (%)	214 (41%)	53 (31%)	83 (41%)	54 (51%)	24 (51%)	
BMI [kg/m^2^]	27.7 ± 4.8	22.9 ± 1.8	27.4 ± 1.4	31.9 ± 1.5	37.6 ± 2.4	<0.001*
Comorbidities						
Arterial hypertension, n (%)	368 (70%)	95 (56%)	142 (70%)	92 (87%)	39 (83%)	<0.001*
Diabetes mellitus type II, n (%)	50 (10%)	8 (5%)	18 (9%)	13 (12%)	11 (23%)	0.003*
Prior stroke, n (%)	42 (8%)	17 (10%)	15 (7%)	6 (6%)	4 (9%)	0.61
CAD, n (%)	100 (19%)	31 (18%)	39 (19%)	22 (21%)	8 (17%)	0.84
OSA, n (%)	33 (6%)	4 (2%)	9 (4%)	12 (11%)	8 (17%)	<0.001*
CHA_2_DS_2_-VASc-score	2.5 ± 1.6	2.5 ± 1.5	2.5 ± 1.6	2.4 ± 1.6	2.9 ± 1.6	0.40

*CB: Cryoballoon-, RF: radiofrequency-pulmonary vein isolation, n: number, AF: atrial fibrillation, BMI: body mass index,* CAD: coronary artery disease, OSA: obstructive sleep apnea.

### Procedural characteristics

#### CB-PVI vs. RF-PVI

As expected, procedural characteristics differ between CB-PVI and RF-PVI. The procedure time was significantly shorter for CB-PVI compared to RF-PVI in the overall study population (CB 75 [60-100] min vs. RF 120 [110-180] min, *p*<0.001). Of note, in 39% of the RF procedures a simultaneous ablation of the cavotricuspid isthmus was performed. As expected, contrast medium volume was higher in the CB group than in the RF group, especially in patients with sOb (all patients: CB 50 [40-80] ml vs. RF 20 [20-30] ml, *p*<0.001; sOb: CB 70 [54.5-102.5] ml vs. RF 20 [15-30] ml, *p*<0.001). Similarly, a lower fluoroscopy dose was detected in the RF group compared to the CB group due to the use of 3D mapping systems (all patients: CB 392.4 [197.9-955.9] μGy x m^2^ vs. RF 282.5 [139.8-507.2] μGy x m^2^, *p*<0.001). The fluoroscopy time was lower in the RF group as well (all patients: CB 14.2 [10-19.1] minutes vs. RF 12 [7.3-18.3] minutes, *p*=0.007).

#### CB-PVI and BMI

A more detailed analysis within the CB-PVI group comparing normal weight with sOb revealed a trend toward longer procedure times in sOb patients (BMI<25 70 [60-90] min vs. BMI≥35 85 [60-120] min; *p*=0.08). The required amount of contrast medium was significantly larger in sOb patients compared to normal weight patients (BMI<25 50 [38-72.5] ml vs. BMI≥35 70 [54.5-102.5 ml] (*p*=0.005)). Similarly, a higher fluoroscopy dose was detected in sOb patients (BMI<25 270.7 [146.2-489.5] μGy x m^2^ vs. BMI≥35 1182.8 [446.6-1946.0] μGy x m^2^ (*p*<0.001)). Of note, there was also a nonsignificant increase in the number of freezes, the freeze duration and the TTI of PVs in patients with a higher BMI (number of freezes: BMI<25 kg/m^2^ 5 [4–6] vs. BMI≥35 kg/m^2^ 6 [5–7] freezes, freeze duration: BMI<25 kg/m^2^ 913.5 [780-1080] s vs. BMI≥35 kg/m^2^ 1112 [869.5-1412] s, TTI: BMI<25 kg/m^2^ 44 [30-75] s vs. BMI≥35 kg/m^2^ 60 [36-85] s).

#### RF-PVI and BMI

The procedural characteristics assessed in the RF group (procedure duration, fluoroscopy dose and amount of contrast medium) as well as the number of impulses, impulse duration and impulse energy did not differ significantly between the different BMI groups, except for the fluoroscopy dose, which was higher in sOb patients compared to normal weight patients (BMI<25 208.9 [111.6-362.8] μGy x m^2^ vs. BMI≥35 646.7 [372.0-918.0] μGy x m^2^, *p*<0.001).

The procedural characteristics are shown in Table 3.

**Table 3:** Procedural characteristics, continuous data are summarized as median (interquartile range), BMI [kg/m^2^]

Procedural characteristics		All		CB		RF		***p*-value** (CB vs. RF)
		n	Median (IQR)	n	Median (IQR)	n	Median (IQR)	
Procedure time [min]	**All**	526	90 (70-120)	320	75 (60-100)	206	120 (110-180)	<0.001*
	**BMI<25**			99	70 (60-90)	71	120 (105-180)	<0.001*
	**BMI 25-30**			119	80 (60-105)	84	130 (100-175)	<0.001*
	**BMI 30-35**			71	75 (60-90)	35	120 (120-150)	<0.001*
	**BMI≥35**			31	85 (60-120)	16	120 (120-180)	<0.001*
	***p*-value** **(BMI)**				0.28		0.97	
Contrast medium [ml]	**All**	404	45 (25-70)	305	50 (40-80)	99	20 (20-30)	<0.001*
	**BMI<25**			94	50 (38-72.5)	32	20 (18.5-28.8)	<0.001*
	**BMI 25-30**			112	50 (35-75.8)	44	20 (20-30)	<0.001*
	**BMI 30-35**			69	50 (40-72.5)	18	20 (18-26.3)	<0.001*
	**BMI≥35**			30	70 (54.5-102.5)	5	20 (15-30)	<0.001*
	***p*-value** **(BMI)**				0.008*		1.0	
Fluoroscopy dose [μGy x m^2^]	**All**	526	340.1 (175.9-719.5)	320	392.4 (197.9-995.9)	206	282.5 (139.8-507.2)	<0.001*
	**BMI<25**			99	270.7 (146.2-489.5)	71	208.9 (111.6-362.8)	0.10
	**BMI 25-30**			119	492.7 (212.8-492.7)	84	273.2 (128.8-460.4)	<0.001*
	**BMI 30-35**			71	361.1 (207.8-880.0)	35	399.0 (246.7-747.1)	1.0
	**BMI≥35**			31	1182.8 (446.6-1946.0)	16	646.7 (372.0-918.0)	0.28
	***p*-value** **(BMI)**				<0.001*		<0.001*	

*statistically significant *p*-value.

*CB: Cryoballoon-, RF: radiofrequency-pulmonary vein isolation, BMI: body mass index.*

The complication rate did not show significant differences between the BMI groups (all patients: 1,7%, NW 1,1%, sOb 2,1%).

### AF recurrence

The mean follow-up time was 356.8±53.3 days, and in 480 out of 526 (91%) patients, a complete 12-month follow-up was available. Follow-up did not differ between both groups (294/320, 92% CB and 186/106, 90% RF). Three patients had died of non-procedure-related causes, and 43 patients were lost to follow-up.

After a 12-month follow-up, the overall freedom from any arrhythmia was high and was comparable for CB-PVI and RF-PVI, regardless of BMI (CB 77% vs. RF 75%, *p*=0.63). No statistically significant correlation of the BMI group and ablation technique with the recurrence rate could be observed in these patients (Table 4). However, sOb patients revealed a trend toward higher AF recurrence rates after RF-PVI as compared to CB-PVI (AF recurrence rate in BMI≥35: CB-PVI 6/25, 24% vs. RF 7/14, 50%, *p*=0.099). Due to the limited number of severely obese patients, this trend was not statistically significant.

**Table 4:** AF recurrence depending on BMI [kg/m^2^]

	CB	RF	*p value*
BMI<25, n (%)	24 (25%)	16 (27%)	0.82
BMI≥35, n (%)	6 (24%)	7 (50%)	0.10
*p value*	0.9179	0.0894	

*CB: Cryoballoon-, RF: radiofrequency-pulmonary vein isolation, BMI: body mass index, n: number.*

As expected, analysis of AF recurrence rates based on AF history revealed significant differences for PAF and pers AF patients in CB- and RF-PVI: CB-PVI (PAF 19%, pers AF 29%, *p*=0.037) and RF-PVI (PAF 17%, pers AF 36%, *p*=0.005), as expected (Table 5).

**Table 5:** AF recurrence, PAF (paroxysmal AF), Pers AF (persistent AF)

	CB	RF	*p value*
PAF + Pers AF, n (%)	67 (23%)	46 (25%)	0.63
PAF, n (%)	33 (19%)	19 (17%)	0.77
Pers AF, n (%)	34 (29%)	27 (36%)	0.35
*p value*	0.037*	0.0046*	

*statistically significant *p*-value.

*CB: Cryoballoon-, RF: radiofrequency-pulmonary vein isolation, PAF: paroxysmal atrial fibrillation, Pers AF: persistent atrial fibrillation, n: number.*

## Discussion

### Main findings

The main findings of this analysis comparing CB-PVI and RF-PVI for different BMI categories are:

1. CB-PVI and RF-PVI are equally safe and effective, regardless of the BMI.
2. In severely obese patients, the procedure time is significantly shorter in CB-PVI, but the amount of contrast medium as well as the fluoroscopy dose is higher compared to RF-PVI.
3. Of note, in patients with BMI≥35 kg/m^2^ there is a trend toward a higher freedom of AF after CB-PVI as compared to RF-PVI. However, the overall freedom from AF is similar after CB- and RF-based PVI.

#### Procedural characteristics

Obesity is a worldwide problem, and the number of obese patients is expected to increase in the next years [20]. Moreover, obesity is not only an independent risk factor for the development of AF [4], but it is also a risk factor for intra- and peri-procedural complications such as aspiration, hypoxemia, hypotension, challenging deep sedation management and infection of groin hematoma [8–13, 22]. Therefore, previous studies have shown that BMI has an influence on procedure time for interventional/surgical procedures and may alter the risk for subsequent complications [11–13, 21, 22].

In this retrospective comparison of 526 de novo PVI using either CB or RF in patients representing all BMI groups, the procedure time for CB-PVI was significantly shorter compared to RF-PVI. A longer procedure time is possibly associated with more obesity-related complications, so the shorter procedure duration with CB-PVI as compared to RF-PVI is potentially beneficial, especially in sOb patients, despite the higher volume of contrast medium and fluoroscopy dosage required. In our experience, with increasing training of investigators, the quantity of contrast medium as well as the fluoroscopy dose may be reduced to a comparable level to RF-PVI.

#### BMI-related findings

**Fig. 2:**
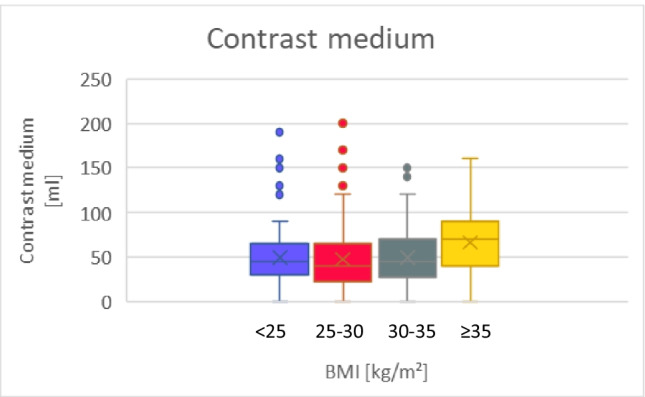
Box plot of the observed procedural characteristics in CB-PVI and RF-PVI depending on BMI. BMI: body mass index

When performing CB-PVI in Ob and sOb patients, repetitive freezes and longer TTI were necessary to achieve complete PVI. For each freeze more contrast medium is needed. Extensive use of contrast medium potentially impairs renal function. Since the amount of contrast medium required increases significantly with higher BMI, renal function should be monitored closely in patients with Ob and sOb [8]. Importantly, the fluoroscopy dose also increases significantly with higher BMI (Fig. 2), regardless of the PVI technique (CB and RF). The higher fluoroscopy dose required in obese patients is harmful for the investigator [23]. Therefore, not only adequate radioprotection but also careful patient selection is crucial.

#### Outcome after CB-PVI vs. RF-PVI

The overall analysis after 12-month follow-up showed that 367/480 patients (76%) were in stable sinus rhythm. Independent of the AF type (PAF or pers AF), the comparison of CB-PVI vs. RF-PVI showed similar rates of freedom from AF. These outcome data are comparable to earlier published trials such as the FIRE and ICE trial [5]. Of note, in the latter trial only PAF patients were included, and the recurrence rate was 35% after a mean follow-up FU of 1.5 years [5].

#### Outcome and BMI

Previous studies have already shown higher AF recurrence rates after RF-PVI with increasing BMI [24, 25]. Despite these previous reported data, our analysis did not reveal significant differences with respect to AF recurrence after CB-PVI or RF-PVI regardless of BMI. However, in sOb patients CB-PVI seems to be more effective as compared to RF-PVI (freedom of AF recurrence CB 76%, RF 50%, *p*=0.099). While this difference is not statistically significant due to the sample size, it suggests a trend toward higher freedom of AF after CB-PVI in sOb patients. Further studies are needed to corroborate these findings. Hence, the shorter procedure time as well as the lower AF-recurrence rate after CB-PVI is potentially beneficial in obese patients to minimize procedure and BMI-related risks with a favorable outcome. However, the increased contrast medium volume and fluoroscopy dose should be considered, especially in patients with comorbidities.

### Limitations

This study is a large single-center study of retrospective nature. The number of sOb patients was relatively small; therefore, some trends did not reach statistical significance. All follow-up data were obtained using medical history and 24-hour Holter, so asymptomatic episodes of AF have potentially been missed in the absence of continuous rhythm monitoring. However, this large cohort reflects a real-world scenario of patients undergoing de novo PVI.

## Conclusion

Data comparing CB-PVI with RF-PVI focusing on BMI are scarce. The present study demonstrates that CB-PVI and RF-PVI have comparable safety and efficacy in obese patients. CB-PVI provides a significantly shorter procedure time. Hence, CB-PVI may minimize possible obesity-related complications (e.g., aspiration, apnea and infections). However, the higher contrast medium volume and fluoroscopy dose required must be considered when performing CB-PVI. Furthermore, in severely obese patients CB-PVI might result in a higher freedom of AF rate. Randomized trials are needed to evaluate the long-term outcome in patients with higher BMI comparing CB-PVI vs. RF-PVI and possible obesity-related complications to corroborate our findings. This issue will become increasingly relevant in the next years due to the rapidly increasing number of patients with AF along with obesity and other comorbidities undergoing PVI.
